# Mechanisms Involved in the Stimulatory and Inhibitory Effects of 5-Hydroxytryptamine on Vagal Mechanosensitive Afferents in Rat Lung

**DOI:** 10.3389/fphys.2022.813096

**Published:** 2022-03-21

**Authors:** You Shuei Lin, Chun-Chun Hsu, Ting Ruan, Lu-Yuan Lee

**Affiliations:** ^1^ Department of Physiology, School of Medicine College of Medicine, Taipei Medical University, Taipei, Taiwan; ^2^ Graduate Institute of Medical Sciences College of Medicine, Taipei Medical University, Taipei, Taiwan; ^3^ School of Respiratory Therapy College of Medicine, Taipei Medical University, Taipei, Taiwan; ^4^ Division of Pulmonary Medicine Department of Internal Medicine, Taipei Medical University Hospital, Taipei, Taiwan; ^5^ School of Medicine, Fu Jen Catholic University, Taipei, Taiwan; ^6^ Department of Physiology University of Kentucky Medical Center, Lexington, KY, United States

**Keywords:** rapidly adapting receptor, slowly adapting receptor, airway, inflammation, bronchoconstriction, salbutamol

## Abstract

Mechanosensitive vagal afferents in the lung, rapidly and slowly adapting receptors (RARs and SARs, respectively), play an important role in eliciting the reflexes that regulate the normal airway function. A profound bronchoconstrictive effect of 5-hydroxytryptamine (5-HT) has been extensively reported in various animal species, but its influence on the SAR and RAR activity is not known. This study investigated the effect of 5-HT on these receptors, and the possible mechanisms involved. Single-fiber activities of these afferents were measured in anesthetized, open-chest, and mechanically ventilated rats. Our results showed that intravenous injection of 5-HT evoked a consistent and pronounced stimulation of phasic RARs. In contrast, 5-HT generated an inconsistent and paradoxical action on SARs: no effect in 29% (5 of 17) of the SARs; stimulation in 35% (6 of 17); and inhibition in the remainder. These responses of both RARs and SARs to 5-HT were reproducible and dose-dependent. After the injection of a high dose of 5-HT (16 μg/kg), the receptor responses slowly reached a peak (after ∼8 s) and returned toward the baseline in ∼20 s, accompanied by a consistent increase in total pulmonary resistance and a decrease in dynamic lung compliance in a temporal pattern very similar to the increased receptor activity. When these changes in lung mechanics induced by 5-HT were prevented by pretreatment with salbutamol, a β_2_ adrenergic receptor agonist, the delayed responses of both RARs and SARs to 5-HT were also abolished, except that the immediate stimulatory effect on a subset of RARs, the silent RARs, was not affected. In conclusion, 5-HT generated a delayed stimulatory effect on RARs and a paradoxical effect on SARs, which resulted primarily from the 5-HT-induced changes in mechanical properties of the lung.

## Introduction

5-hydroxytryptamine (5-HT) is a monoamine formed by decarboxylation of essential amino acid tryptophan, stored in secretory granules, and released from platelets and mast cells upon degranulation. Its diverse and complex pharmacological properties and physiological actions (Adnot et al., 2013), and the significant role as an autacoid and chemical mediator in the pathogenesis of allergic airway inflammatory diseases such as asthma have been well recognized (Cazzola and Matera, 2000; Luo et al., 2021). In addition, a profound bronchoconstrictive effect of 5-HT on both central and peripheral airways has been reported in various animal species (Macquin-Mavier et al., 1991; Cazzola et al., 1995; Szarek et al., 1995).

In a recent study, we reported that intravenous (i.v.) injection of 5-HT exerted an intense stimulatory effect on bronchopulmonary C-fibers, the chemosensitive vagal afferents innervating the lung and airways. This stimulatory effect was mediated through activation of the 5-HT_3_ receptor because it was completely blocked by pretreatment with tropisetron (Trop), a selective antagonist of the 5-HT_3_ receptor. In that study, we also observed another interesting response: when the conduction of the C-fiber afferents was selectively blocked by perineural capsaicin treatment of both cervical vagus nerves in anesthetized, spontaneously breathing rats, the same 5-HT injection consistently evoked an augmented breath (Hsu et al., 2019), a vagal-mediated respiratory reflex response that is believed to be elicited by activation of the rapidly adapting receptors (RARs) in the lung (Glogowska et al., 1972; Lee and Yu, 2014).

RARs and slowly adapting receptors (SARs) are the major types of mechanosensitive receptors located in the lung and airways (Sant’Ambrogio, 1982; Coleridge and Coleridge, 1986; Lee and Yu, 2014; Yu, 2021). Their sensory signals are conducted by myelinated afferent fibers in the vagus nerves and project to the respiratory centers located in the medulla. These receptors are activated by changes in volume, flow, and pressure in the respiratory tract, and therefore sensitive to changes of the mechanical properties of the lung/airways (Sant’Ambrogio, 1982; Coleridge and Coleridge, 1986; Lee and Yu, 2014). Furthermore, a subset of RARs is also sensitive to various inhaled irritant substances and endogenous chemical mediators (Sellick and Widdicombe, 1971; Sant’Ambrogio and Widdicombe, 2001; Lee and Yu, 2014). Together, they play an important role in eliciting the reflexes that regulate the rate and depth of breathing, bronchomotor tone, airway secretion, and other important cardiorespiratory functions; for example, stimulation of RARs can cause cough, mucous hypersecretion, and bronchoconstriction; and stimulation of SARs has been shown to induce airway dilation, inhibit expiratory muscle activity and elicit cardiovascular reflex responses (e.g., sinus arrhythmia, peripheral vasodilation) (WIDDICOMBE, 1981; Coleridge and Coleridge, 1986; Lee and Yu, 2014).

Despite the important background information described above, whether and how 5-HT altered the activities of these mechanoreceptors in the lung remained to be determined. The present study was carried out to answer this question, and to investigate the possible mechanisms involved in the action of 5-HT on these receptors.

## Methods

The experimental procedures described below were in accordance with the recommendation in Guide for the Care and Use of Laboratory Animals published by the National Institutes of Health and approved by the Institutional Animal Care and Use Committees of both University of Kentucky and Taipei Medical University.

### Animal Preparation

Male Sprague-Dawley (SD) rats were anesthetized with intraperitoneal injection of α-chloralose (100 mg/kg) and urethane (500 mg/kg). The supplemental doses were administrated by i. v. injection to maintain the elimination of pain reflexes produced by pinching the rat’s tail. The trachea was cannulated *via* a tracheotomy. The right femoral artery was cannulated for measuring arterial blood pressure (ABP). The right jugular vein was cannulated for the administration of anesthetics and pharmacological agents. Body temperature was maintained at ∼36°C throughout the experiments using a servo-controlled heating pad placed under the rat lying in a supine position. Animals were euthanized by i. v. injection of an overdose of KCl (150 mg/kg) at the end of the experiment.

### Measurement of Single-Fiber Activity of RARs and SARs

Single-unit activities of vagal pulmonary afferents were recorded following the protocol described in our previous study in rats (Ho et al., 2001). Briefly, the activity of single-unit pulmonary myelinated afferents was recorded from open-chest and mechanically ventilated (f = 50 breaths/min; V_T_ = 8–10 ml/kg) rats. The right cervical vagus nerve was separated from the carotid artery, sectioned rostrally, and placed in a small dissecting platform and immersed in a pool of mineral oil. Signals of the action potential were amplified, monitored by an audio monitor and displayed on an oscilloscope. The thin filament was further split until the fiber activity (FA) arising from a single unit was electrically isolated.

To eliminate the possibility of activating RARs and SARs by reflex bronchoconstriction, the left cervical vagus nerve was also sectioned. The afferent activity of RARs and SARs were searched initially by their distinct phasic discharge synchronous with the respiratory cycles of the ventilator, and by their responses to hyperinflation of the lung (tracheal pressure [P_tr_] = 30 cmH_2_O) and lung deflation (P_tr_ = 0 cmH_2_O), each maintained at a constant level for 10 s. RARs and SARs were then further identified by their adaptation indexes (AIs) measured by the response to a constant-pressure lung inflation (P_tr_ = 30 cmH_2_O, 10 s); AI was calculated in each fiber by dividing the difference in FA between the first 2 s during the constant-pressure lung inflation by the FA of the first second, and expressed as a percentage (Widdicombe, 1954). Fibers with AIs of >80% and <50% were classified as RARs and SARs, respectively. The fibers with AIs between 50 and 80% were identified as “intermediate type” receptors (Bergren and Peterson, 1993; Yu, 2020); they were of a small number and not included in this study. RARs were further divided into two subgroups: phasic and silent; the latter had either no or irregular baseline activity. The general location of each receptor was determined by its responses to a gentle pressing of the lung lobes and airways with a wet cotton swab at the end of each experiment. Signals of action potential, P_tr_ and ABP were digitized and analyzed by a data-acquisition system and computer software (Biocybernetics TS-100, Taipei, Taiwan).

### Measurement of Lung Mechanics

Rats were anesthetized, vagotomized and mechanically ventilated as described above. Transpulmonary pressure was measured as the difference between the P_tr_ and intrapleural pressure (0 cmH_2_O in the open-chest preparation). Airway flow was measured and integrated to give tidal volume. Transpulmonary pressure, respiratory flow and integrated volume were analyzed on a breath-by-breath basis for total pulmonary resistance (R_L_) and dynamic lung compliance (C_dyn_) by the same computer software (Biocybernetics TS-100).

### Experimental Protocols

**Study series 1**: to study the effect of i. v. bolus injections of 5-HT on RARs and its dose dependency, the responses of RARs to increasing doses of 5-HT (4, 8, 16 μg/kg), with a 15-min interval between injections, were determined. **Study series 2**: to study the mechanism(s) involved in the stimulatory effect of 5-HT, the responses of RARs to the highest dose of 5-HT (16 μg/kg) were determined before and 15 min after pretreatment with Trop (15 μg/kg), a selective antagonist of 5-HT_3_ receptor; and also before and 10 min after pretreatment with salbutamol (Sal; 0.5 mg/kg), a β_2_ adrenergic receptor agonist. **Study series 3**: to investigate the possible effect of 5-HT on lung mechanics and to test the reproducibility, the responses of R_L_ and C_dyn_ to i. v. bolus injection of 5-HT (16 μg/kg) were determined before, 15 min after pretreatment with Trop (15 μg/kg), and 10 min after Sal (0.5 mg/kg). **Study series 4**: to characterize the effects of i. v. bolus injection of 5-HT (16 μg/kg) on silent RARs. **Study series 5**: to study the mechanism(s) involved, the initial and delayed responses of silent RARs to 5-HT (16 μg/kg) were determined before, 15 min after pretreatment with Trop (15 μg/kg), and 10 min after Sal (0.5 mg/kg). **Study series 6**: the responses of SARs to increasing doses of i. v. injections of 5-HT (4, 8, 16 μg/kg) were determined following the same protocol described for RARs in Study series 1. **Study series 7**: the effects of Trop and Sal pretreatments on the SAR responses to i. v. injection of 5-HT (16 μg/kg) were determined following the same protocol described for RARs in Study series 2.

### Statistical Analysis

Results were analyzed with the one-way repeated-measures analysis of variance (ANOVA). When ANOVA showed a significant positive interaction, pair-wise comparisons were made with a *post-hoc* analysis (Fisher’s least significant difference). A value of *p* < 0.05 was considered significant. Data are reported as means ± SEM.

## Results

A total of 72 vagal afferents innervating the lung and airways (22 phasic RARs, 21 silent RARs and 29 SARs) were studied in 64 rats (body weight 365–410 g). These receptors were located in all four lobes of the right lung; with the largest number in the middle lobe and smallest in the accessory lobe. Because of the small dimension of the rat lung structure and the limited space available for probing the extrapulmonary airways in our preparation, the difference in receptor distribution between lung parenchyma and intrapulmonary airways was not determined in this study. The receptors that had been studied but their locations could not be identified in the lung or airways were excluded from the data analysis.

### Study Series 1: Stimulatory Effect of 5-HT on Phasic RARs

**FIGURE 1:**
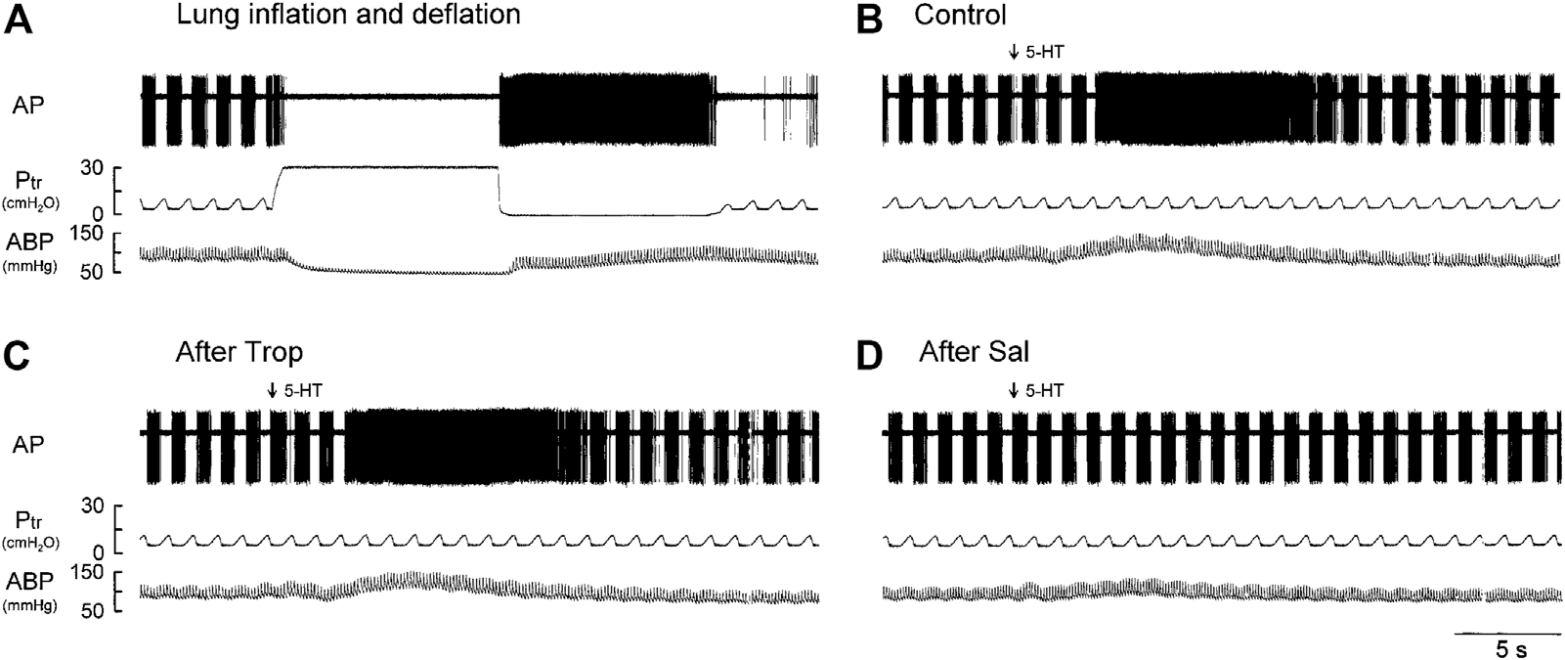
Experiment records illustrating the responses of a phasic RAR located in the right lower lobe in an anesthetized, vagotomized, open-chest, and mechanically ventilated rat (405 g) to: **(A)** lung inflation generated by a constant P_tr_ held at 30 cmH_2_O for 10 s, followed immediately by lung deflation (P_tr_ = 0 cmH_2_O, held for 10 s); **(B–D)** i. v. injections of 5-HT (16 μg/kg) before, 15 min after pretreatment with Trop (15 μg/kg), and 10 min after pretreatment with Sal (0.5 mg/kg), respectively. 5-HT (0.1 ml) was first slowly injected into the catheter and then flushed (marked by arrow) as a bolus with vehicle (isotonic saline, 0.3 ml). At least 15 min elapsed between two injections of 5-HT to avoid possible tachyphylaxis. AP, action potential; P_tr_, tracheal pressure; ABP, arterial blood pressure.

Phasic RARs discharged synchronously with the ventilator cycles, and their activity was present during the expiratory phase in all the phasic RARs recorded in this study; among them, 42% of the receptors discharged continuously into the early inspiratory phase (e.g., Figure 1). The phasic RARs responded sharply and vigorously to lung inflation, but declined rapidly or ceased discharge within the first 1–2 s when the lung inflation was maintained at a constant P_tr_ of 30 cmH_2_O (AI > 80%; Figure 1A). During the prolonged (10 s) lung deflation, the FA increased further and did not show any adaptation to the continuous deflation of the lung (Figure 1A).

**FIGURE 2:**
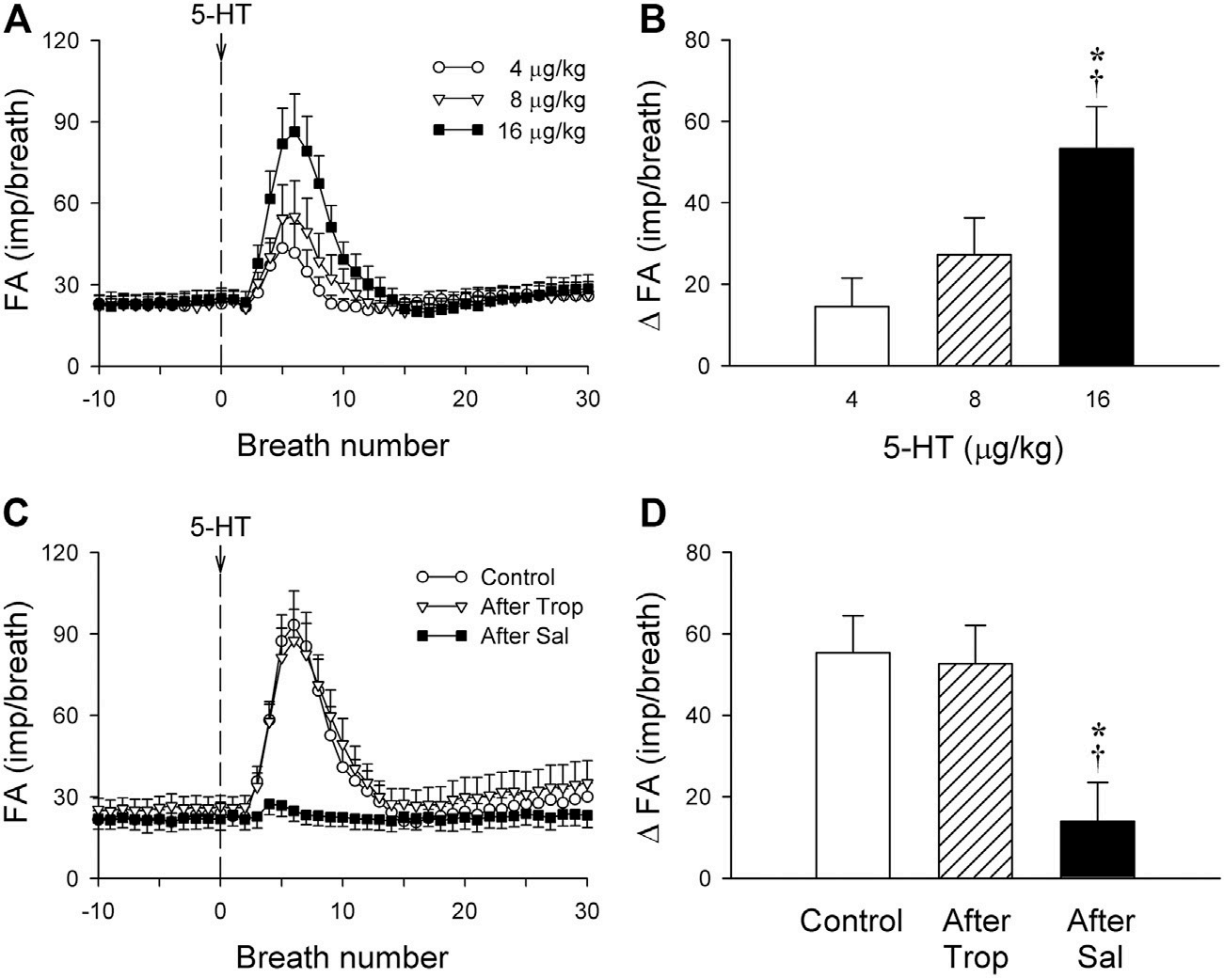
**(A)** Responses of phasic RARs (n = 12) to i. v. injections (at the vertical dashed line) of increasing doses of 5-HT (4, 8 and 16 μg/kg), separated by 15-min interval between injections, illustrated the dose-dependence of the stimulatory effect of 5-HT in anesthetized, vagotomized, open-chest, and mechanically ventilated rats. Fiber activity (FA) was measured on a breath-by-breath basis. **(B)** The increase in FA (ΔFA) was the difference in FA between the baseline (20-breath average) and the peak response (average of 5 consecutive breaths) that emerged within the first 20 breaths after the 5-HT injection. * and †, significantly different (*p* < 0.05) from the responses to 4 and 8 μg/kg of 5-HT, respectively. **(C,D)** Effect of 5-HT (16 μg/kg) on fiber responses of phasic RARs (n = 10) before, 15 min after pretreatment with Trop (15 μg/kg), and 10 min after pretreatment with Sal (0.5 mg/kg). * and †, significantly different (*p* < 0.05) from the control response and the response after pretreatment with Trop, respectively. Data are means ± SEM.

Intravenous bolus injection of 5-HT stimulated all phasic RARs tested (e.g., Figure 1B). The increased FA did not emerge until after a latency of 3–8 s (after the injection), reached a peak in 6–11 s (average 8.4 s or 7 breaths after the 5-HT injection), and returned toward the baseline in ∼20 s (Figures 1B,2A). During this delayed stimulation, the phasic discharge pattern continued in these RARs, and the pronounced increase in FA occurred during both inspiratory and expiratory phases. The average peak responses of these receptors (n = 12) to increasing doses of 5-HT exhibited a dose-dependent pattern (Figures 2A,B).

Intravenous bolus injection of 5-HT also caused a transient increase in ABP (89.3 ± 3.2 and 132.4 ± 4.7 mmHg before and after the injection, respectively; *p* < 0.05, n = 29), but no change in heart rate was found.

### Study Series 2: Effects of Trop and Sal on 5-HT-induced Activation of Phasic RARs

The baseline FA of phasic RARs was not affected significantly by pretreatment with Trop or Sal (control: 22.4 ± 3.4 impulses/breath; after Trop: 24.5 ± 4.1 impulses/breath, *p* > 0.05, n = 12; after Sal: 21.9 ± 3.9 impulses/breath, *p* > 0.05, n = 12) (Figure 2C). However, the peak response of phasic RARs to the highest dose of 5-HT (16 μg/kg) was significantly attenuated by Sal, but not affected by Trop (ΔFA during control: 55.4 ± 8.6 impulses/breath; after Sal: 13.9 ± 9.8 impulses/breath, *p* < 0.05, n = 12; after Trop: 52.6 ± 9.2 impulses/breath, *p* > 0.05, n = 12) (Figures 1C,D, 2C,D).

### Study Series 3: Effects of Trop and Sal on 5-HT-induced Changes in Lung Mechanics

**FIGURE 3:**
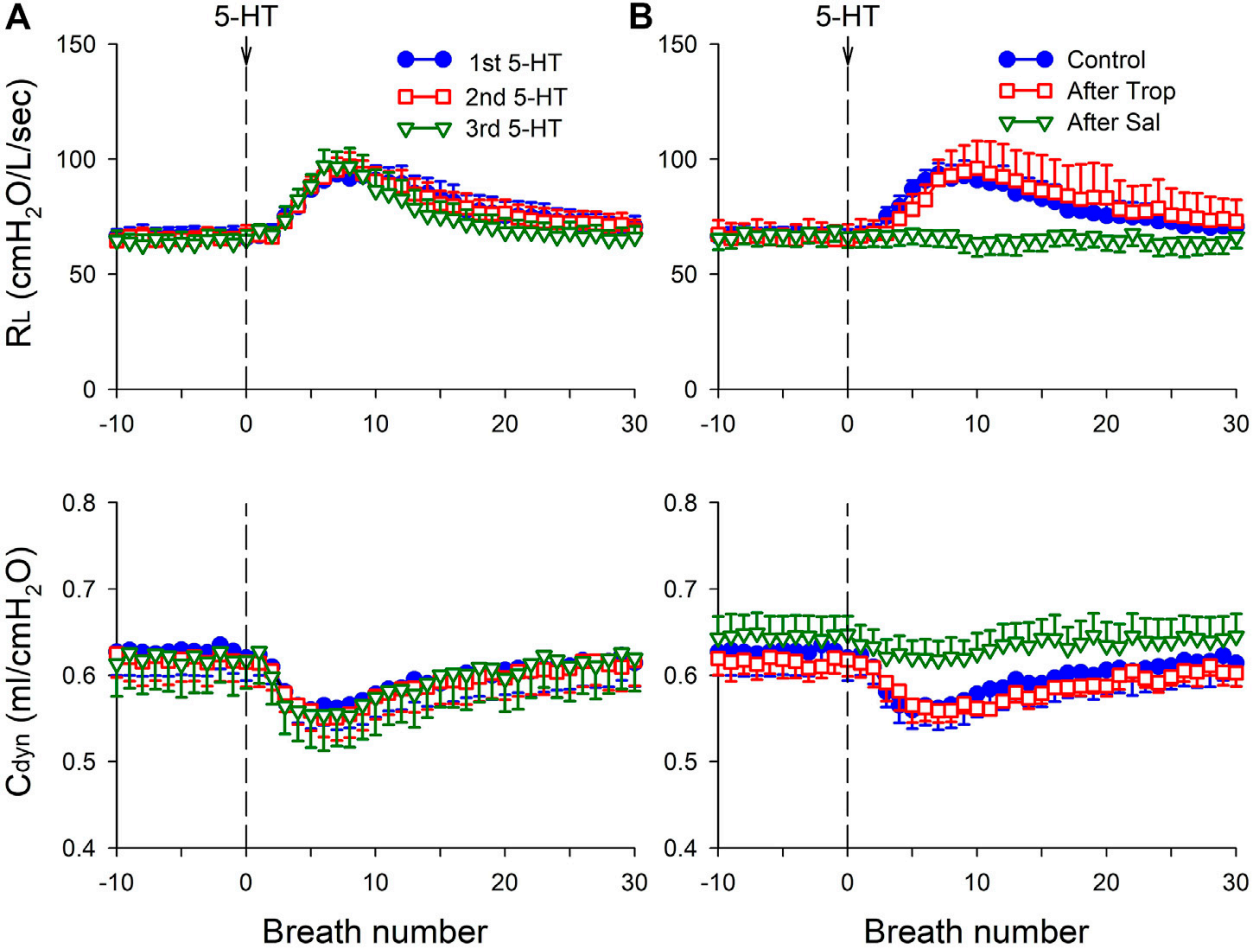
**(A)** Group data illustrating the reproducibility of the effects of i. v. injection of 5-HT (16 μg/kg) on total pulmonary resistance (R_L_) and dynamic lung compliance (C_dyn_) in eight anesthetized, vagotomized, open-chest, and mechanically ventilated rats; at least 15 min elapsed between two injections of 5-HT. **(B)** Responses of R_L_ and C_dyn_ to the 5-HT injections (16 μg/kg) before, 15 min after pretreatment with Trop (15 μg/kg), and 10 min after pretreatment with Sal (0.5 mg/kg) in the same eight rats as in (A).

Intravenous bolus injections of the highest dose of 5-HT induced an increase in R_L_ and a decrease in C_dyn_ in rats (n = 8; Figure 3A). These changes in lung mechanics began in 3–7 s (after the injection), reached a peak in 9–13 s, and lasted for 28–37 s (Figure 3A). The changes in R_L_ and C_dyn_ were reproducible when the same 5-HT injection was repeated 20–40 min later in the same rats (Figure 3A). The changes in R_L_ and C_dyn_ were not affected after pretreatment with Trop, but were completely prevented by pretreatment with Sal. The peak increase in R_L_ (ΔR_L_) in response to the 5-HT (16 μg/kg) injection was 25.2 ± 4.3 (cmH_2_O/L/s) at control; 29.0 ± 9.3 (cmH_2_O/L/s) after Trop (n = 8, *p* > 0.05); and 0.21 ± 4.80 (cmH_2_O/L/s) after Sal (n = 8, *p* < 0.05); the largest decrease in C_dyn_ (ΔC_dyn_) in response to the same 5-HT injection was 0.07 ± 0.03 (ml/cmH_2_O) at control; 0.06 ± 0.02 (ml/cmH_2_O) after Trop (n = 8, *p* > 0.05); and 0.03 ± 0.02 (ml/cmH_2_O) after Sal (n = 8, *p* < 0.05) (Figure 3B).

### Study Series 4: Stimulatory Effect of 5-HT on Silent RARs

**FIGURE 4:**
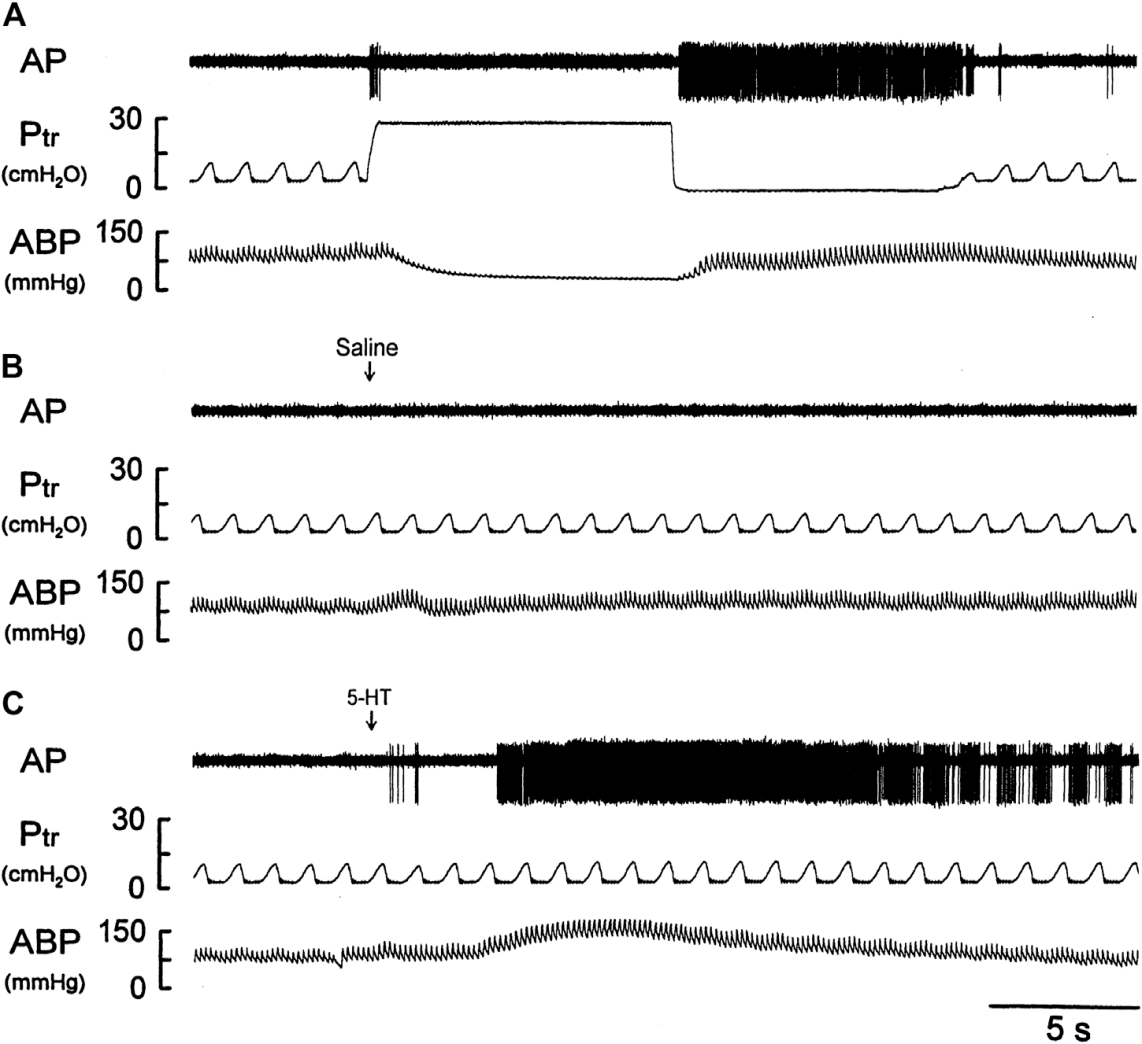
Experimental records illustrating the responses of a silent RAR located in the right middle lobe to **(A)** a constant-pressure lung hyperinflation (P_tr_ = 30 cmH_2_O) maintained for 10 s and followed by lung deflation (P_tr_ = 0 cmH_2_O) for 10 s; **(B,C)** i. v. bolus injection of vehicle (isotonic saline) and 5-HT (16 μg/kg), respectively, in an anesthetized, vagotomized, open-chest, and mechanically ventilated rat (375 g). See the legend of Figure 1 for detailed explanations.

**FIGURE 5:**
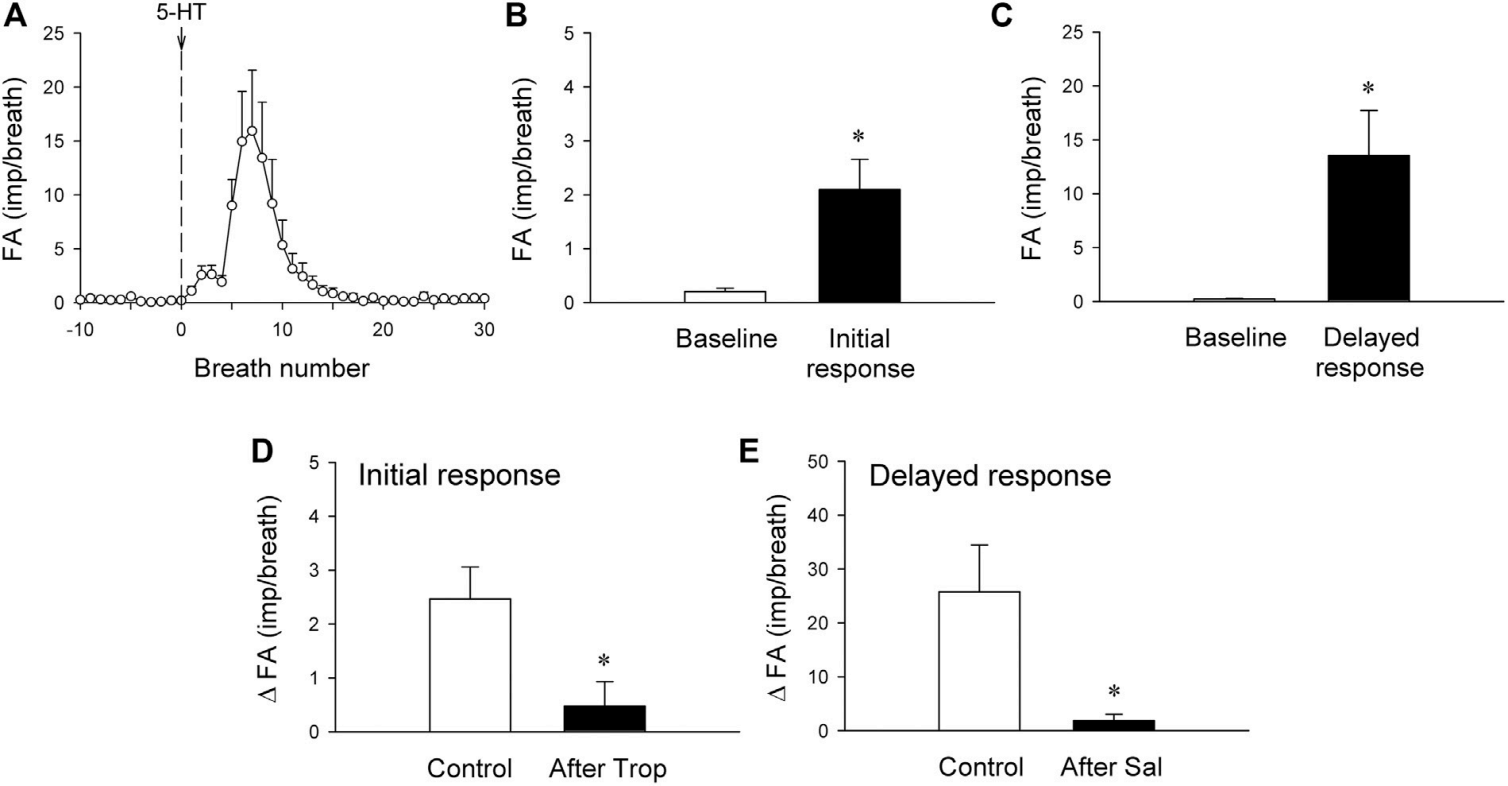
**(A)** histogram showing average responses of silent RARs to i. v. bolus injection of 5-HT recorded from 21 fibers in 18 anesthetized, vagotomized, open-chest, and mechanically ventilated rats. Fiber activity (FA), measured on a breath-by-breath basis, revealed that the responses of silent RARs to the i. v. injection of 5-HT (16 μg/kg; vertical line) consisted of two distinctly separate phases: the initial **(B)** and delayed phases **(C)** (see experimental record in Figure 4C). ΔFA for the initial response was measured as the difference between the baseline FA (20-breath average) and the peak FA (average of the first 3 breaths occurring after the 5-HT injection); ΔFA for the delayed response as the difference between the baseline FA (20-breath average) and the peak FA (average of 5 consecutive breaths) during the first 4–20 breaths. **(D)** The initial response to 5-HT before and 15 min after pretreatment with Trop (15 μg/kg; n = 6 from 6 rats). **(E)** The delayed response to 5-HT before and 10 min after pretreatment with Sal (0.5 mg/kg; n = 6 from 5 rats). *, significantly different (*p* < 0.05) from the baseline in **(B,C)** or control response in **(D,E)**. Data are means ± SEM.

There was either no or very low baseline (before 5-HT) activity in silent RARs (0.18 ± 0.03 impulses/breath) (Figures 4A–C). Intravenous bolus injection of 5-HT (16 μg/kg) activated 16 of 21 silent RARs recorded in 18 rats, and their discharges were found in two distinct phases: a short burst of activity was evoked immediately (<1 s) after the 5-HT injection (e.g., Figure 4C), but not after injection of vehicle (isotonic saline; e.g., Figure 4B); and a delayed response began to emerge at 4–9 s after the 5-HT injection (Figures 4C,5A). In the 16 silent RARs activated by 5-HT, 11 responded to 5-HT during the initial phase (Figure 5B), 13 during the delayed phase (Figure 5C), and eight during both initial and delayed phases (e.g., Figure 4C). Interestingly, during the delayed stimulation, the increase in FA in these silent RARs exhibited a sustained (tonic) discharge and did not show a clear phasic pattern.

### Study Series 5: Effect of Trop and Sal on 5-HT-induced Activation of Silent RARs

The immediate stimulatory effect of 5-HT on silent RARs was significantly reduced after the pretreatment with Trop: ΔFA = 2.5 ± 0.6 impulses/breath and 0.5 ± 0.4 impulses/breath, before and after Trop, respectively (*p* < 0.05, n = 6; Figure 5D).

The delayed stimulatory effect of 5-HT on silent RARs was almost completely abolished after the pretreatment with Sal: ΔFA = 25.8 ± 6.3 impulses/breath and 1.9 ± 0.9 impulses/breath, before and after Sal, respectively (*p* < 0.05, n = 6; Figure 5E).

### Study Series 6: Paradoxical Effects of 5-HT on SARs

**FIGURE 6:**
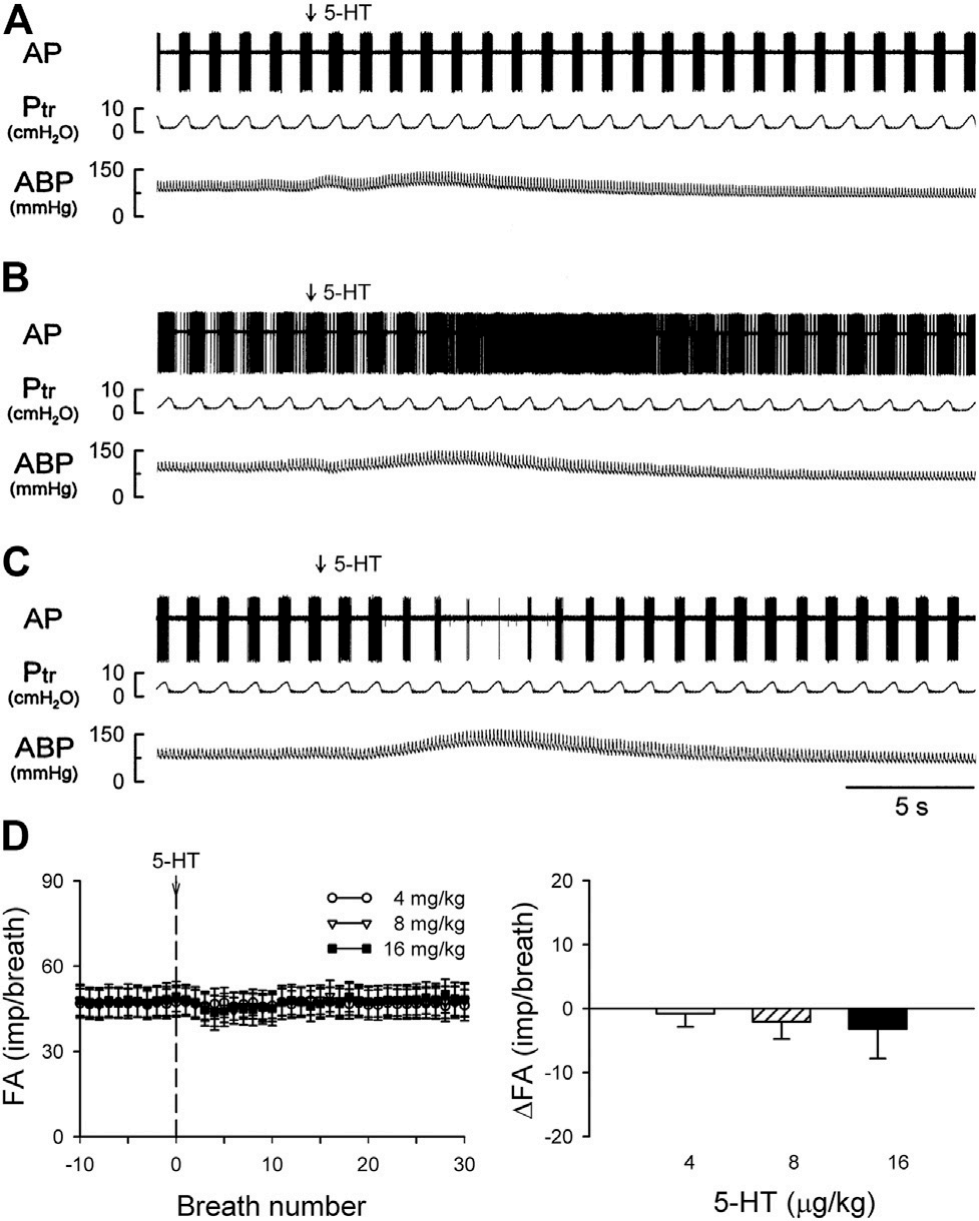
**(A–C)** experimental records illustrating three different types of SAR responses to i. v. injections of 5-HT (16 μg/kg) in three anesthetized, vagotomized, open-chest, and mechanically ventilated rats: **(A)** no effect of 5-HT (405 g; receptor in right lower lobe); **(B)** delayed stimulation (400 g; right middle lobe); **(C)** delayed inhibition (395 g; right lower lobe). **(D)** Average responses of the entire group of SARs (n = 17) to i. v. bolus injection of increasing doses of 5-HT (depicted by the vertical dashed line), separated by a 15-min interval between two injections. FA: fiber activity; ΔFA was calculated as the difference between the baseline FA (20-breath average) and the peak FA (average of 5 consecutive breaths). Data are means ± SEM.

SARs discharged synchronously with the ventilator cycles, and their activity was present predominantly during the inspiratory phase (Figures 6A–C). These receptors responded sharply to lung inflation and continued to discharge when the lung inflation was maintained at a constant P_tr_ of 30 cmH_2_O (AI < 50%). During the prolonged (10 s) lung deflation, SARs either ceased discharge completely or their phasic activity was markedly reduced.

**FIGURE 7:**
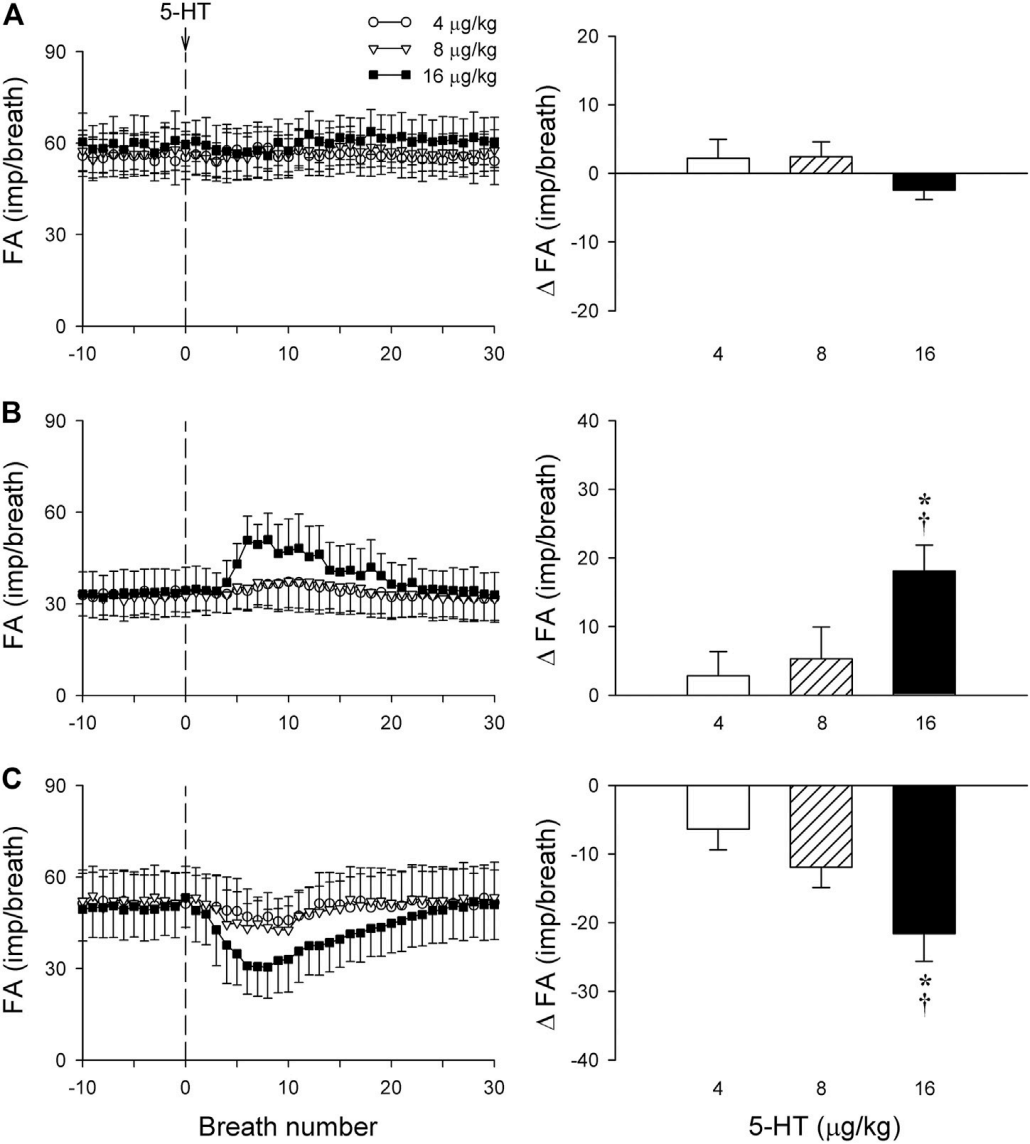
The same group of SARs in Figure 6 (n = 17) were divided into three subgroups based upon their different types of responses to 5-HT: **(A)** no response to 5-HT (n = 5); **(B)** delayed stimulation (n = 6); **(C)** delayed inhibition (n = 6). Only one SAR was studied in each animal. Left panels: histograms comparing the FA responses to i. v. injection of increasing doses of 5-HT (4, 8 and 16 μg/kg); right panels: peak responses of FA (ΔFA) to the same three doses of 5-HT injections. * and †, significantly different (*p* < 0.05) from the responses to 4 and 8 μg/kg of 5-HT, respectively. Data are means ± SEM. See the legend of Figure 2 for detailed explanations.

Intravenous bolus injection of 5-HT (16 μg/kg) caused paradoxical effects on the 17 SARs tested in this study: no effect (n = 5; e.g., Figure 6A), delayed stimulation (n = 6; e.g., Figure 6B), and delayed inhibition (n = 6; e.g., Figure 6C). The 5-HT injection did not generate any significant change in FA when the data of all 17 SARs were pooled as a group (Figure 6D). However, when these SARs were divided into three subgroups based upon their responses described above, both significant stimulatory and inhibitory effects of the 5-HT injection were clearly revealed in the two subgroups, each with a latency of 3–7 s and reaching the peak response in 7–10 s (after the injection) (Figures 7B,C).

### Study Series 7: Effect of Trop and Sal on 5-HT-induced Stimulation and Inhibition of SARs

**FIGURE 8:**
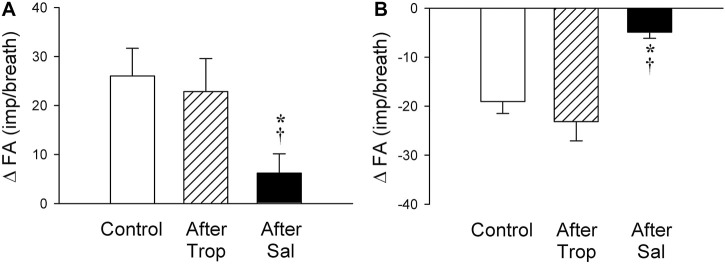
Effect of pretreatments with Trop (15 μg/kg) and Sal (0.5 mg/kg) on the afferent responses to i. v. injections of 5-HT (16 μg/kg) in two subgroups of SARs selected based upon their individual responses to 5-HT, as described in Figure 7: **(A)** stimulation (n = 6); **(B)** inhibition (n = 6). Only one SAR was studied in each animal. * and †, significantly different (*p* < 0.05) from the control response and the response after pretreatment with Trop, respectively. Data are means ± SEM.

The baseline FA of SARs was slightly reduced after the pretreatment with Sal, but not affected by Trop (control: 45.2 ± 2.3 impulses/breath; after Trop: 44.2 ± 2.5 impulses/breath, *p* > 0.05; after Sal: 41.8 ± 2.9 impulses/breath; *p* < 0.05). However, both types of responses, stimulation and inhibition, were significantly attenuated after the pretreatment with Sal in their respective subgroups of SARs (*p* < 0.05, n = 6 and *p* < 0.05, n = 6, respectively) (Figures 8A,B). In contrast, these responses were not affected by the pretreatment with Trop (Figures 8A,B).

## Discussion

We identified and classified RARs and SARs in the rat lung based upon the standard definitions and criteria of the “AI” that have been established by previous investigators. In the open-chest, mechanically ventilated rats, the baseline activities of both SARs and phasic RARs were synchronized with the respiratory cycles of the ventilator, and responded vigorously to lung inflation and deflation, respectively, resembling the characteristic features of these receptors described by other investigators in larger mammalian species such as dogs, cats, and rabbits (Yu 2005; Lee and Yu, 2014).

Results of this study showed that i. v. injection of 5-HT generated profound effects on both major types of mechanoreceptors in the rat lung: a consistent and pronounced stimulation of phasic RARs but a paradoxical action on SARs. In both cases, the responses of these receptors to 5-HT were reproducible and dose-dependent. After the injection of the high dose of 5-HT (16 μg/kg), the receptor responses reached a peak slowly (∼8 s after the injection) and returned toward the baseline in ∼20 s (Figures 2, 5, 7). The receptor response was accompanied consistently by an increase in R_L_ and a decrease in C_dyn_; the temporal pattern of these changes (Figure 3) closely mirrored that of the increase in receptor activity. When these changes in lung mechanics induced by 5-HT were prevented by the administration of Sal (Figure 3B), the delayed responses of both RARs and SARs to 5-HT were nearly completely abolished (Figures 2C,D,5E,8A,8B), except that the immediate response in the subset of RARs, the silent RARs, was not affected. Thus, these results clearly indicated an important role of the change in mechanical properties of the lung in the delayed responses of RARs and SARs to 5-HT.

Intravenous injection of 5-HT evoked both initial and delayed stimulatory effects on the silent RARs (Figures 4, 5). These two distinctly separate responses were mediated through two different transduction mechanisms. The immediate response was completely prevented by pretreatment with Trop, a selective antagonist of the 5-HT_3_ receptor. In addition, considering the rapid onset (<1 s; e.g., Figure 4) of the response, these observations collectively suggest that the activation of RARs was probably mediated through the direct action of 5-HT on the 5-HT_3_ receptors. Indeed, a similar immediate stimulatory effect on 5-HT_3_ receptors has also been observed in the response of vagal bronchopulmonary C-fiber endings to i. v. injection of 5-HT in our recent study (Hsu et al., 2019). In sharp contrast, the delayed effect was not affected by Trop, but abolished by pretreatment with Sal. Although the immediate response was relatively mild in magnitude, compared to the delayed response (e.g., Figures 4, 5), it was reproducible in the same receptors between different trials and consistent between different silent RARs tested. This observation of the dual-modality, both chemical and mechanical, of these silent-RARs in response to the 5-HT challenge further exemplified the unique characteristics of the afferent properties of this subset of RARs (Lee and Yu, 2014).

Despite the relatively scarce number, these silent RARs were consistently found in the rat lung, and their presence has also been identified in other species (Sant’Ambrogio, 1982), such as dogs (Kou and Lee, 1990, 1991), cats (Sellick and Widdicombe, 1971), rabbits (Lin et al., 2011), guinea pigs (Bergren and Sampson, 1982), and mice (Lin et al., 2017). Their afferent properties and reflex functions remain ill-defined and elusive. The abrupt discharge pattern of these RARs in their immediate response to 5-HT (Figures 4, 5) was similar to that of pulmonary C-fibers (Hsu et al., 2019). The firing behavior of these silent RARs also exhibited certain features resembling the “high-threshold Aδ vagal afferents” described by Yu and coworkers in rabbits (Lin et al., 2011) though distinct responses to lung hyperinflation and deflation were found in these silent RARs but not in the high-threshold Aδ receptors. These silent RARs in rats also exhibited transduction properties similar to the chemosensitive RARs in larger mammalian species (Sellick and Widdicombe, 1971; Sampson and Vidruk, 1975; Matsumoto, 1989; Kou and Lee, 1990, 1991; Sant’Ambrogio and Widdicombe, 2001); for example, they displayed a mild sensitivity to chemical stimulants of C-fibers (e.g., acid, capsaicin, smoke) and did not exhibit clear phasic discharge during eupneic mechanical ventilation. In addition, we cannot rule out the possibility that some of these silent RARs are the “cough receptors” that have been identified by previous investigators in the tracheobronchial tree of other species such as dogs (Widdicombe, 1954) and guinea pigs (Canning et al., 2004).

How the delayed stimulation of both silent and phasic RARs (e.g., Figures 1, 2, 4, 5) was generated by 5-HT could not be clearly delineated in this study. However, as illustrated in Figure 3, i. v. injection of 5-HT induced both an increase in R_L_ and a decrease in C_dyn_, and both these changes in the mechanical properties of the lung are known to act as effective stimuli of RARs (Sampson and Vidruk, 1975; Sant’Ambrogio, 1982; Kou and Lee, 1991; Lee and Yu, 2014). Specifically, previous investigators have established convincing evidence indicating that a decrease in regional lung compliance (or an increase in elastic recoil) was a potent stimulator of RARs (Yu et al., 1987; Pisarri et al., 1990). This finding was further supported by the observations that RARs were consistently activated when a mild increase in interstitial fluid volume or pressure in the lung was experimentally induced by pulmonary venous congestion or pulmonary lymphatic obstruction, leading to a reduction in lung compliance (Hargreaves et al., 1991; Kappagoda and Ravi, 2006). Furthermore, i. v. injection of 5-HT is known to induce pulmonary hypertension by constricting arterioles and post-capillary venules, which can promote fluid filtration from the pulmonary capillary into the interstitial space (Brigham and Owen, 1975; Bhattacharya et al., 1982). In addition, airway smooth muscle contraction induced by 5-HT can result in the closure of small-diameter peripheral airways and lead to local atelectasis and decreased regional lung compliance, which in turn can stimulate RARs.

The bronchoconstrictive effect of 5-HT has been well documented in various species, including rats; it can be generated by a direct action of 5-HT on the 5-HT_2_ receptor present on smooth muscles (Martin et al., 1994; Cazzola et al., 1995; Szarek et al., 1995) or an indirect mechanism *via* the secondary release of acetylcholine from parasympathetic efferent endings (Macquin-Mavier et al., 1991; Szarek et al., 1995) and/or other chemical mediators (Cazzola et al., 1995), which causes the airway smooth muscle contraction. In a small number of rats (n = 3) carried out in the same manner as in the present study, we found that pretreatment with atropine (0.1 mg/kg, i. v.), a cholinergic muscarinic antagonist, did not abolish either the bronchoconstriction or the delayed stimulation of phasic RARs following the 5-HT injection (16 μg/kg, i. v.; unpublished data), suggesting a relatively minor contribution of the possible secondary release of acetylcholine. The stimulatory effect of bronchoconstriction on RARs has also been demonstrated extensively and is considered a major contributing factor to the RAR excitatory response to a wide range of inhaled chemical irritants (e.g., cigarette smoke, ammonia, ether) (Bergren and Sampson, 1982; Sant’Ambrogio and Widdicombe, 2001) and various endogenous inflammatory mediators (e.g., histamine, leukotrienes) (Bergren and Sampson, 1982; Sant’Ambrogio and Widdicombe, 2001). Although the precise transduction mechanism is not yet known, a possible involvement of increasing airway wall tension generated by smooth muscle contraction has been postulated (Kou and Lee, 1991; Yu, 2005; Lee and Yu, 2014).

When all the SARs studied were treated as a single group, their overall response did not show any significant effect of 5-HT in this study (Figure 6). However, distinctly opposite responses were found among these SARs when they were separated into subgroups based upon their responses: 5-HT caused a stimulatory effect in 35% (6 of 17) of these SARs; an inhibitory effect in 35% (6 of 17); and no effect in the remainder (Figures 6, 7). We don’t know the exact mechanism(s) underlying these seemly paradoxical responses, but a possible contributing factor should be considered. It was demonstrated in an interesting study by Sato et al. (1993) that bronchoconstriction induced by i. v. injection of methacholine resulted in a pronounced uneven distribution of ventilation as a result of the inhomogeneity of impedance and time constant (the product of resistance and compliance) in different regions of the lung in anesthetized, open-chest, and mechanically ventilated dogs. In the present study, rats were prepared in a similar experimental condition: anesthetized, open-chest, and mechanically ventilated. It seems conceivable that the bronchoconstriction caused by the 5-HT injection may have induced a similar uneven distribution of ventilation in the rat lung. If so, a stimulatory effect of 5-HT on those SARs may reflect that the receptors were located in the lung region receiving a proportionally larger distribution of the tidal volume delivered by the ventilator during inspiration, whereas an inhibitory effect was found on the SARs located in the lung region with a smaller volume expansion. The fact that these opposite responses to 5-HT between these two subgroups of SARs were significantly diminished by the pretreatment with Sal (Figure 8) is consistent with this hypothesis. To further test this possible mechanism, we carried out follow-up experiments in a small number of SARs (n = 3) that exhibited a delayed inhibitory response to 5-HT injection. An injection of methacholine (5 μg/kg, i. v.) also evoked a delayed inhibitory effect in a pattern very similar to that of 5-HT on the same SARs; these data are submitted with this manuscript as supplemental materials. Similarly, in the SARs that a delayed stimulatory effect was generated by 5-HT injection, the injection of methacholine also induced a similar stimulatory effect. These observations have provided further support to our hypothesis.

The “intermediate type” of vagal mechanoreceptors in the lung, identified by their AIs in the range of 50–80%, may have mechanosensitive properties that are different from SARs and RARs (Bergren and Peterson, 1993; Yu, 2020). Because these intermediate receptors were encountered in a small number and not included in this study, the possible effects of 5-HT on these receptors are not known.

In conclusion, 5-HT exerts a delayed but profound stimulatory effect on both silent and phasic types of RARs in rat lungs. These effects on RARs are probably caused indirectly by the 5-HT-induced changes in mechanical properties of the lung. 5-HT also evoked an immediate stimulation of the silent RARs, which is mediated through direct activation of the 5-HT_3_ receptors located on the sensory nerve terminals in these RARs. Given the fact that the increase of the 5-HT level in the lung is closely correlated with the severity of airway inflammatory diseases and the decline of pulmonary functions (Cazzola and Matera, 2000; Luo et al., 2021), a possible involvement of these stimulatory effects of 5-HT on these mechanosensitive lung afferents in the manifestation of various symptoms and pathophysiological changes of lung functions should be recognized and considered.

## Data Availability

The original contributions presented in the study are included in the article/supplementary material, further inquiries can be directed to the corresponding author.
